# Mathematical modeling reveals differential dynamics of insulin action models on glycerol and glucose in adolescent girls with obesity

**DOI:** 10.3389/fphys.2022.895118

**Published:** 2022-08-05

**Authors:** Griffin S. Hampton, Kai Bartlette, Kristen J. Nadeau, Melanie Cree-Green, Cecilia Diniz Behn

**Affiliations:** ^1^ Department of Applied Mathematics and Statistics, Colorado School of Mines, Golden, CO, United States; ^2^ Division of Pediatric Endocrinology, University of Colorado Anschutz Medical Campus, Aurora, CO, United States; ^3^ Ludeman Center for Women’s Health Research, University of Colorado Anschutz Medical Campus, Aurora, CO, United States

**Keywords:** glycerol, glucose, insulin, insulin resisitance, lipolysis, mathematical model

## Abstract

Under healthy conditions, the pancreas responds to a glucose challenge by releasing insulin. Insulin suppresses lipolysis in adipose tissue, thereby decreasing plasma glycerol concentration, and it regulates plasma glucose concentration through action in muscle and liver. Insulin resistance (IR) occurs when more insulin is required to achieve the same effects, and IR may be tissue-specific. IR emerges during puberty as a result of high concentrations of growth hormone and is worsened by youth-onset obesity. Adipose, liver, and muscle tissue exhibit distinct dose-dependent responses to insulin in multi-phase hyperinsulinemic-euglycemic (HE) clamps, but the HE clamp protocol does not address potential differences in the dynamics of tissue-specific insulin responses. Changes to the dynamics of insulin responses would alter glycemic control in response to a glucose challenge. To investigate the dynamics of insulin acting on adipose tissue, we developed a novel differential-equations based model that describes the coupled dynamics of glycerol concentrations and insulin action during an oral glucose tolerance test in female adolescents with obesity and IR. We compared these dynamics to the dynamics of insulin acting on muscle and liver as assessed with the oral minimal model applied to glucose and insulin data collected under the same protocol. We found that the action of insulin on glycerol peaks approximately 67 min earlier (*p* < 0.001) and follows the dynamics of plasma insulin more closely compared to insulin action on glucose as assessed by the parameters representing the time constants for insulin action on glucose and glycerol (*p* < 0.001). These findings suggest that the dynamics of insulin action show tissue-specific differences in our IR adolescent population, with adipose tissue responding to insulin more quickly compared to muscle and liver. Improved understanding of the tissue-specific dynamics of insulin action may provide novel insights into the progression of metabolic disease in patient populations with diverse metabolic phenotypes.

## Introduction

The obesity epidemic now affects a significant portion of the world, causing insulin resistance and metabolic dysregulation in multiple organs of the body. The worldwide prevalence of overweight and obesity has approximately doubled from 1980 to 2015, affecting adults and children of all ages, and is forecasted to reach levels over 50% by 2030 (Kelly et al., 2008; Chooi et al., 2019). The metabolic syndrome as defined in the National Health and Nutrition Examination Survey (NHANES) is related to insulin resistance (IR) and shows an increased risk for developing type 2 diabetes and cardiovascular disease. The metabolic syndrome was calculated to affect 34.7% of the U.S. population in 2016, with a significant increase in the incidence in young adults from 2011 to 2016 (Aguilar et al., 2015; Hirode and Wong, 2020). Related to this obesity and metabolic dysfunction, approximately 34.2 million adults in the United States have type 2 diabetes (T2D) (Centers for Disease Control and Prevention, 2020), and among youth the incidence rate of T2D is also increasing and expected to quadruple from 2010 to 2050 (Imperatore et al., 2012; Mayer-Davis et al., 2017; American Diabetes, 2020). Of grave concern, T2D appears to be much more aggressive in youth than in adults, including poor response to interventions effective in adults, and early onset of diabetes complications (RISE Consortium and Investigators, 2019; Group et al., 2021; Utzschneider et al., 2021). Even when dysglycemia is already present, adolescents secrete much higher concentrations of insulin than adults, likely driven by their marked IR (RISE Consortium, 2018; Utzschneider et al., 2020). This high morbidity and the unique physiologic features of insulin sensitivity and secretion in youth drive the necessity to specifically investigate the systems involved in metabolic disease development in youth. By better understanding the unique pathology of metabolic disease in youth, better treatments can be developed and personalized for individuals.

Metabolic dysregulation often arises from an imbalance in energy consumption and expenditure. During fasting, energy is primarily provided from energy stored in adipose and hepatic tissue. In a healthy individual, when energy is acquired through ingesting food, the mechanisms that provide endogenous energy sources are suppressed, so that the ingested fuel can be used and stored. Insulin facilitates the transition from an endogenous to exogenous energy source, and it manages glycerol, free fatty acid (FFA), and glucose systems across different metabolic states. In addition to suppressing the release of glucose from the liver and stimulating glucose uptake in hepatic and peripheral tissues (Petersen and Shulman, 2018), insulin is the most potent antilipolytic hormone: it suppresses lipolysis, and reduces the use of FFA as an energy source. IR is defined as a decreased biological response to insulin, which leads to increased insulin secretion, eventually causing pancreatic β-cell failure and T2D (Ronald Kahn, 1978; Arner, 2002; Cree-Green et al., 2019a). IR is tissue specific, and it may manifest in individual tissues at different points in disease progression. It is hypothesized that the development of IR in adipose tissue, resulting in excess circulating FFA and glycerol, may induce IR in other tissues (Arner and Rydén, 2015). Elevated FFA concentrations may contribute to dysglycemia in multiple ways, including impairing β-cell insulin secretion and vascular function, and directly inducing hepatic and skeletal muscle IR (Arner, 2001; Arner, 2002; Arner and Rydén, 2015; Sondergaard et al., 2017), thereby emphasizing the importance of characterizing adipose IR.

The gold standard in assessing insulin action on adipose tissue is a low dose hyperinsulinemic euglycemic (HE) clamp with stable isotope tracers. The HE clamp determines the steady state concentration of insulin, that is, necessary to suppress FFA and/or glycerol release into circulation. Using different insulin infusion rates as part of a multi-step clamp with glucose and glycerol tracers, the insulin sensitivity of adipose, liver, and peripheral tissue can be determined (Conte et al., 2012). While effective at quantifying some aspects of adipose health, the HE clamp is resource intensive and narrow in application as it relies on steady state values produced from glucose and insulin infusions rather than the coordinated physiologic response that occurs with oral nutrient ingestion (Sondergaard et al., 2017). Moreover, the HE clamp does not provide insight into the dynamics of insulin action on adipose, liver, or muscle tissue. An insulin-modified frequently sampled intravenous glucose tolerance test (IM-FSIVGTT) is a dynamic test where glucose is administered intravenously followed by an insulin bolus, showing metabolic dynamics under non-physiologic circumstances. An oral glucose tolerance test (OGTT) is a more physiologically complete dynamic test where participants ingest glucose orally through a sugary drink, allowing for the contribution of multiple gut hormones that may also play a role in the coordinated response to nutrition. Therefore, to focus on the dynamic response of adipose, liver, and muscle tissue to insulin under a more physiologic state, we quantify the dynamics of insulin action on glycerol and glucose during an oral glucose tolerance test (OGTT).

Both glycerol and FFA are released during lipolysis, but glycerol is a better marker of lipolysis due to differences in recycling between glycerol and FFA. FFA can either be released from adipose cells into the bloodstream or be recycled within adipose cells in a process by which the FFA are reincorporated into triacylglycerides and absorbed by neighboring cells prior to entry to the bloodstream (Coppack et al., 1999; Landau, 1999; Reshef et al., 2003; Wolfe and Chinkes, 2005; Magkos et al., 2012; Cree-Green et al., 2016; Cree-Green et al., 2019a; Cree-Green et al., 2019b). The process of intracellular and intratissue recycling complicates the dynamics of FFA and must be considered when evaluating adipose metabolism with FFA. In contrast, because adipose tissue lacks the expression of glycerol kinase (Steinberg et al., 1961), glycerol is not recycled in adipose tissue as it cannot be reincorporated into triacylglycerides. Instead, circulating glycerol produced by lipolysis is taken up primarily by the liver *via* hepatic glycerol kinase expression, allowing glycerol to be phosphorylated and reincorporated into triacylglycerides (Coppack et al., 1999; Jensen, 1999; Landau, 1999). The absence of local glycerol recycling in adipose makes glycerol an appealing metabolite to track adipose metabolism. Whereas lipolysis from adipose tissues is the primary source for intravascular glycerol, a small proportion of glycerol is also produced *via* glycogenolysis and gluconeogenesis (Rotondo et al., 2019). These synthetic processes are regulated by glycerol-3-phosphate phosphatase and phosphoglycolate phosphatase which control the amount of glycerol made by glycogenolysis in the fasting state, and then gluconeogenesis in the fed state (Possik et al., 2022). It is estimated that up to 10%–15% of intravascular glycerol during prolonged fasting may be attributed to these processes, but the proportion attributed in the fed state is not as clear. The fasting contribution from glycogenolysis is higher with long fasting durations. In our study, participants had a monitored fast of 12 h, so the contribution from glycogenolysis is expected to be low. The contribution from gluconeogenesis is related to serum glucose concentrations. As none of our participants had diabetes, the contribution from this pathway is also expected to be low. Therefore, we consider changes in glycerol concentration to primarily reflect insulin-mediated changes in lipolysis.

Mathematical models of glucose metabolism have contributed a fundamental understanding of interactions in glucose and insulin dynamics (Ajmera et al., 2013; Cobelli et al., 2014). These models describe how insulin induces glucose uptake by peripheral tissue and reduces glucose production from endogenous sources under different experimental conditions, and the Oral Minimal Model (OMM) describes glucose dynamics during an OGTT (Bergman et al., 1979; Bergman, 1989; Dalla Man et al., 2002; Ha et al., 2016; Bartlette et al., 2021). Although insulin concentrations may be modeled directly (Bergman RNB et al., 1981; Picchini et al., 2005; Ramos-Roman et al., 2012; Ha et al., 2016), an intermediate variable of insulin action is often introduced to account for the delay between changes in insulin concentrations and observed effects on glucose concentrations (Bergman, 1989; Dalla Man et al., 2002), and this delay may increase as insulin sensitivity decreases. The concepts of glucose metabolic modeling have also been extended to other tissues and metabolic systems including adipose tissue (Roy and Parker, 2006; Periwal et al., 2008; Ramos-Roman et al., 2012; Thomaseth et al., 2014; Li et al., 2016; Young and Periwal, 2016). In previous work we modeled glycerol dynamics with an implicit insulin effect on the glycerol rate of appearance that was estimated using glycerol stable isotope tracer data (Diniz Behn et al., 2020). Periwal and colleagues proposed a model of interacting FFA and insulin dynamics to measure adipose metabolism during an IM-FSIVGTT (Periwal et al., 2008). Their model used a Hill function to represent insulin action-dependent lipolysis and described both glucose and FFA dynamics using a single insulin action term, suggesting that the dynamics of insulin action on glucose and FFA were similar in this study. These models have been successfully employed to assess adipose metabolism in translational studies utilizing IVGTTs (Adler-Wailes et al., 2013; Levine et al., 2020).

To characterize the dynamics of orally-stimulated adipose metabolism, we develop a differential-equations based mathematical model that describes the interaction between glycerol and insulin concentrations during an OGTT. We use the modeling infrastructure of existing FFA models as a basis for our glycerol-insulin model, and we explicitly represent the effects of insulin on lipolysis. We apply the glycerol-insulin model and the OMM to OGTT data from a population of obese and overweight adolescent girls with and without polycystic ovary syndrome (PCOS). This population is characterized by a significant degree of IR and metabolic dysregulation (Bartlette et al., 2021; Ware et al., 2022). To quantify tissue-specific insulin action, we compare simulation results and model parameters associated with the glycerol model and the OMM. The differences in the dynamics of insulin action on glycerol and glucose systems were the primary focus of this study.

## Methods

### Participants

The development of the glycerol model and analysis of insulin action dynamics was conducted on data collected in the APPLE (Androgens and Post-Prandial LivEr metabolism: liver and fat regulation in overweight adolescent girls; NCT02157954) study. This study was performed to explore metabolic abnormalities associated with PCOS and develop new adolescent specific models to understand IR. It was approved by the Colorado Multiple Institutional Review Board. All participants provided informed consent if they were 18–21 years old or parental consent and participant assent if they were 12–17 years old.

The participants were recruited for this cross-sectional study from pediatric clinics at Children’s Hospital Colorado. The inclusion criteria were age 12–21 years, female sex, postpubertal Tanner Stage 5 status, at least 18 months post-menarche, and overweight/obese status (BMI  ≥ 90th percentile for age and sex). The participants had a sedentary lifestyle (<3 h routine exercise per week, validated with both a 3-day activity recall and 7-day accelerometer use). The exclusion criteria were a confirmed diagnosis of diabetes (HbA1c ≥ 6.5%), pregnancy, anemia, liver diseases other than non-alcoholic fatty liver disease (NAFLD), an alanine transferase (ALT) level greater than 125 IU/L, and use of medications known to affect insulin sensitivity or glucose metabolism (including systemic steroids and antipsychotics) in the last 6 months. Metformin and oral contraceptives were excluded in all participants except in metformin (*n* = 6) and contraceptive (*n* = 10) sub-cohorts. Participants with PCOS were defined according to the NIH criteria: 1) an irregular menstrual cycle and 2) clinical and/or biochemical evidence of hyperandrogenism (Zawadzki JD, 1992). Total body fat and fat free mass percentages was assessed by standard DEXA methods (Hologic, Waltham, MA).

From the ninety-two studied participants, the population analyzed in this paper was a subset of sixty-six participants (18 with normal menses and forty-eight with PCOS, described in Table 1). Of the ninety-two study participants the following were excluded: Sixteen with missing OGTT time points precluding modeling and 10 participants randomized to receive exanatide during the OGTT, because exenatide is known to alter insulin dynamics.

**TABLE 1:** Population description. These values are reported as population numbers or means ± the standard deviation.

Variable	Values
Physical characteristics	
Number (n)	66
Age (years)	15.6 ± 2
Race (n) White/Black	59/7
Ethnicity (n) Hispanic/non-Hispanic	35/31
Disease State (n) Obese Control/PCOS/PCOS + drug	18/33/15
BMI (kg/m^2^)	35.5 ± 5.7
Weight (kg)	95.8 ± 16.9
Fat Free Mass (kg)	49.6 ± 7.3
Fat Mass (kg)	42.9 ± 10.8
Height (cm)	164.1 ± 7.1
Waist Circumference (cm)	106.5 ± 11.9
Metabolic Characteristics	
6h Insulin Sensitivity (dL/kg/min per μU/mL )	2.9 ± 2.4 × 10^–4^
Fasting glucose (mg/dl)	90 ± 9
2-h glucose (mg/dl)	142 ± 25
Fasting glycerol (μmol/L)	118 ± 26
Fasting FFA ( μmol/L )	625 ± 139
Fasting Insulin ( μU/mL )	26 ± 15
Peak Insulin ( μU/mL )	361 ± 207
Peak Insulin Time (min)	84 ± 47

### Protocol

Each participant had two study-visits: 1) an initial consent/screening for eligibility; 2) an overnight monitored fast during the follicular phase of the menstrual cycle followed by a six-hour OGTT. Before the metabolic study visit, participants refrained from physical activity for 3 days. The afternoon and evening prior to the OGTT, each participant consumed an isocaloric diet (65% carbohydrate, 15% protein, 20% fat). After the evening meal, each participant refrained from activity and followed a monitored inpatient 12-h fast, followed by a frequently sampled OGTT. Baseline fasting metabolite concentrations were determined prior to the OGTT. At 8 a.m., participants ingested 75 g glucose and 25 g of fructose. Fructose was included to distinguish abnormal hepatic fat metabolism. The drink was consumed in a three-minute window at time 0 and blood samples were taken at the following time points: −20, −10, 0, 10, 20, 30, 45, 60, 75, 90, 105, 120, 135, 150, 165, 180, 210, 240, 300, and 360 min. Blood glucose was measured at the bedside with the StatStrip^®^ Hospital Glucose Monitoring System (Novo Biomedical, Waltham, MA, United States). Serum insulin was measured with radioimmunoassay (Millipore, Billerica, MA, United States). Serum glycerol concentrations were obtained from an ELISA assay (R-Biopharm, Washington, MO, United States).

### Oral minimal model for glucose dynamics

**FIGURE 1:**
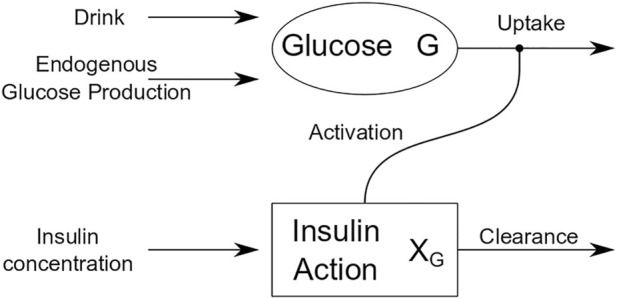
Schematic of oral minimal model (OMM).

OGTT glucose dynamics for each participant were described using the Oral Minimal Model (OMM) (Dalla Man et al., 2002), a one-compartment mathematical model that describes the effect of insulin on glucose and provides an estimate of whole-body insulin sensitivity (S_I_), as reported previously (Bartlette et al., 2021). Figure 1 is a schematic that shows how insulin action affects the uptake term of the glucose dynamics.

The oral minimal model equations are

$$\dot{G}=-\left[S_{G}+X_{G}\right]G+S_{G}G_{b}+\frac{Ra_{meal}}{V}$$ (1)

$$\dot{X_{G}}=\{\begin{array}{cc} -p_{2}^{G}\;X_{G} & ,I\left(t\right)<\;I_{b}\\ -p_{2}^{G}\;X_{G}\;+\;p_{3}\left(I\left(t\right)-\;I_{b}\right) & ,I\left(t\right)\geq \;I_{b} \end{array}$$ (2)

where G(t) is glucose concentration in mg/dL; $X_{G}\left(t\right)$ is insulin action on glucose; I(t) is the insulin concentration; $G_{b}$ and $I_{b}$ are basal glucose and insulin concentrations, respectively; $S_{G}$ is the glucose effectiveness; $p_{2}^{G}$ is a time constant of insulin action; $p_{3}$ is a constants of insulin action clearance and appearance; and $Ra_{meal}\left(\text{α},t\right)$ is a piecewise-linear function describing the rate of appearance of exogenous glucose in the bloodstream. The initial values for the OMM are $G\left(0\right)=G_{b}$ and $X_{G}\left(0\right)=0$ . Six-hour OGTT data from this population were fit to the OMM implemented in SAAM II (SAAM II software v 2.2, The Epsilon group, Charlottesville, VA, United States) as we previously detailed in Bartlette et al. (2021). The parameters we determined in this prior study were used to model the glucose dynamics for all participants in the present study. The insulin action profiles generated from the best-fit parameters were the focus of comparison between insulin-mediated glucose and glycerol dynamics.

### Glycerol dynamics model

**FIGURE 2:**
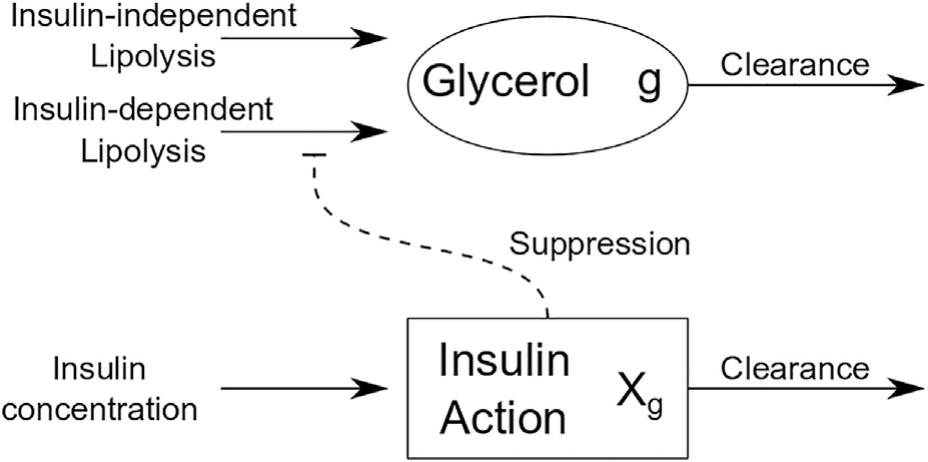
Schematic of glycerol model.

Informed by models of FFA dynamics, we developed a differential equations-based model for glycerol dynamics that utilizes the concept of insulin action as an intermediate variable between measured insulin and its action on adipose tissue. Figure 2 is a schematic of insulin action on glycerol dynamics that illustrates insulin action on glycerol production. By contrast with insulin action’s role to activate glucose uptake in OMM, insulin action in the glycerol model suppresses glycerol production. The equations for the glycerol model are as follows:

$$\dot{g}=-S_{g}g+l_{0}+\frac{l_{2}}{1+{\left(\frac{X_{g}}{X_{2}}\right)}^{A}}$$ (3)

$$\dot{X_{g}}=\{\begin{array}{cc} -p_{2}^{g}\;X_{g} & ,I\left(t\right)<\;I_{b}\\ -p_{2}^{g}\;X_{g}\;+\;p_{2}^{g}\;\left(I\left(t\right)-I_{b}\right) & ,I\left(t\right)\geq \;I_{b} \end{array}$$ (4)

where g(t) is the concentration of glycerol in μ mol/L; $X_{g}\left(t\right)$ is insulin action on glycerol; $p_{2}^{g}$ is a time constant of insulin action; I(t) is the insulin concentration; $I_{b}$ is the basal insulin concentration; $S_{g}$ is the effectiveness of glycerol uptake; $l_{0}$ is the insulin independent lipolysis rate; $l_{2}$ is the insulin dependent (suppressible) lipolysis rate; $X_{2}$ scales insulin action; and A affects how aggressively changes in insulin action result in changes of lipolysis suppression. Lipolysis is modeled as the sum of an insulin independent lipolysis rate, $l_{0}$ , and a Hill function representing insulin action-dependent lipolysis and describing the transition from maximum lipolysis rate, $l_{0}+l_{2}$ , to the minimum lipolysis rate, $l_{0}$ , as insulin action increases. The Hill function is the functional form that was determined to best fit the dynamics of FFA suppression (Periwal et al., 2008).

### Glycerol model fitting process

Before the glycerol model was fit to glycerol data for each participant, the data were truncated to reflect the time period from the drink ingestion (t = 0) to the time at which the participant’s glucose concentration reached a nadir concentration following the glucose excursion induced by the drink. The choice to fit data from t = 0 to the glucose concentration nadir avoided physiological complications due to the high prevalence of reactive hypoglycemia in this population, and it provided a standard check point by which to compare participants. More details are included in the Discussion.

The basal concentration of insulin was determined by averaging the concentrations at timepoints −20, −10, and 0 min. The model was then fit to the truncated data in MATLAB (Mathworks, Natick, MA) using the interior point algorithm FMINCON and the built-in ode solver ODE23S with an absolute tolerance of 1e-10. The FMINCON algorithm minimized an objective function analogous to the objective function described in Periwal et al. (2008); Li et al. (2016). Briefly, this objective function uses single spectrum analysis with only one eigenvalue retained to generate a representative smoothing of the data. Variance of the data is calculated by squaring the standard deviation of the squared difference between the experimental data and the representative smooth curve generated from the single spectrum analysis. The error term is the sum of the square differences between the experimental data and the numeric solution produced by ODE23S divided by the calculated variance. As in previous work, we fixed the parameter A to 2 because the model was not sensitive to this parameter and fixing it improved model identifiability (Li et al., 2016).

Lipolysis parameters were seeded in a physiological range between 0 and approximately 200% of the analogous parameter values reported by Periwal and colleagues (Periwal et al., 2008). The $S_{g}$ and $p_{2}^{g}$ parameters were seeded between 0 and 1. If the initial parameters did not produce a valid model state (i.e., model states were not real or positive), all parameters would be randomly reseeded until the initial model state was valid. For the optimization, all parameters were constrained to be nonnegative and parameters representing proportions, $S_{g}$ and $p_{2}^{g}$ were restricted to range between 0 and 1. The glycerol and insulin concentration data for each participant were fit with FMINCON 75 times. The solution with the lowest objective function value of the 75 runs was selected as the best fit parameter set.

### Analysis of insulin action dynamics

All analysis was done in MATLAB (Mathworks, Natick, MA). To quantify the differences in insulin action dynamics associated with glucose and glycerol, we defined three metrics on the insulin action profiles. The first metric determines the difference in time between the insulin action peak for each metabolite and the peak insulin concentration. The magnitudes of each delay were computed for both glucose and glycerol for all participants and compared with a Wilcoxon signed rank test. The Wilcoxon test was chosen to compare the two distributions because the data are paired and not normally distributed. Since the dynamics of glucose and glycerol come from the same participant, using the same insulin concentrations as a forcing function, the samples are not independent.

The second metric determines the difference in time between the insulin action peak for glucose and the insulin action peak for glycerol. This measure describes the relative timing of insulin action for each metabolite. The difference in timing for glucose and glycerol action was evaluated using a one-sample Student’s t-test to establish if the difference was equal to zero. The third metric determines the difference in the normalized insulin actions at the time point associated with the glucose nadir (i.e., the lowest glucose value after the glucose peak). This measure quantifies the relative strength of insulin on the glucose system compared to the glycerol system at the time of the glucose nadir. To compute this measure, the insulin action curves for each metabolite were normalized by the peak insulin action values, respectively, and then the insulin action values at the time point associated with the glucose nadir were determined. The normalized glycerol insulin action nadir value was subtracted from the normalized glucose insulin action nadir value to obtain the relative difference in insulin actions at the nadir. The relative difference in the normalized insulin actions at the nadir was evaluated with a one-sample Student’s t-test to test if the difference was equal to zero.

In addition to these metrics comparing the insulin action profiles, and we also compared the estimated parameters $p_{2}^{G}$ and $p_{2}^{g}$ that govern the insulin action dynamics for glucose and glycerol, respectively. Qualitatively, larger insulin action time constants reflect smaller delays from the insulin concentration profile while smaller insulin action time constants reflect larger delays from the insulin concentration profile. Since the insulin action time constants have an exponential effect on insulin action, we compared the magnitude of time constant values for each metabolic system using $log_{10}\left(p_{2}^{G}\right)$ and $log_{10}\left(p_{2}^{g}\right).$ The $log_{10}\left(p_{2}^{g}\right)$ and $log_{10}\left(p_{2}^{G}\right)$ parameter distributions were not approximately normal. We compared $log_{10}\left(p_{2}^{G}\right)$ and $log_{10}\left(p_{2}^{g}\right)$ with a Wilcoxon signed rank test.

## Results

### Mathematical modeling of glucose and glycerol dynamics

**FIGURE 3:**
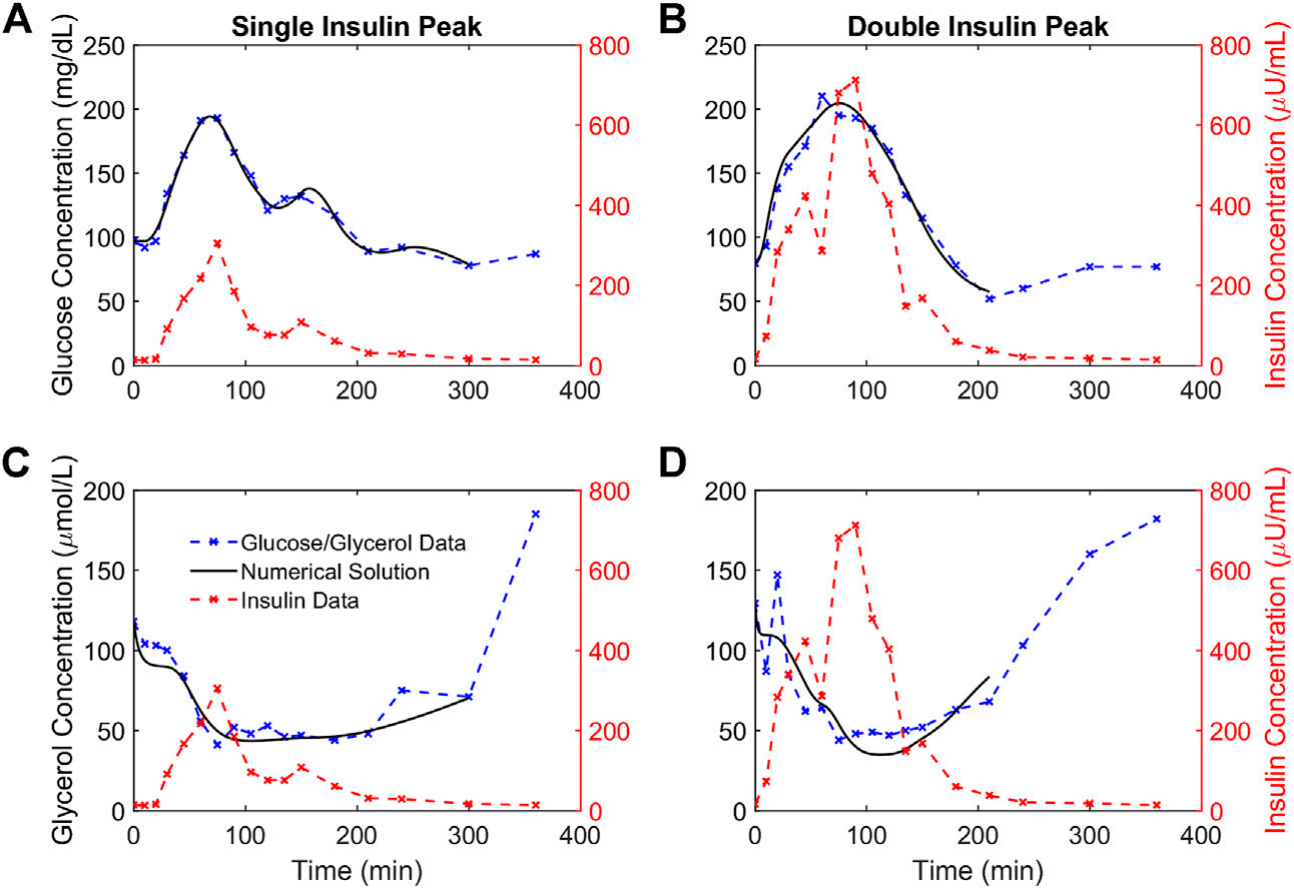
Numerical solutions and OGTT data for glucose and glycerol in two representative participants. **(A,B)**. The numerical solutions for glucose (black) are shown relative to the data (blue) and insulin (red) concentrations for two representative participants demonstrating a single insulin peak **(A)** and a double insulin peak **(B)**, respectively. **(C,D)**. The numerical solutions for glycerol (black) are shown relative to the data (blue) and insulin (red) concentrations for the same representative participants and show the suppression of glycerol concentrations in response to insulin concentrations. The lowest glucose concentration following the glucose excursion is taken to be the end point for the glucose and glycerol numerical solutions for each individual.

For each participant we fit OMM and the glycerol model to OGTT data. Following ingestion of the drink, glucose and insulin concentrations increased and glycerol concentrations decreased for all participants. Although the functional form for insulin action was the same for both models, we found that obtaining good fits to the glucose and glycerol data required separate representations of the dynamics of insulin action on each metabolite. Figure 3 shows the OMM and glycerol model fits to glucose and glycerol dynamics, respectively, for two representative individuals from our cohort. These participants were selected to show different dynamic features associated with varying degrees of glycemic dysregulation in this population. The first participant’s insulin profile has a single insulin peak (SIP). The second participant’s insulin profile has a secondary peak prior to the main peak resulting in a double insulin peak (DIP). The SIP participant reaches peak insulin concentration at 75 min while the DIP participant’s insulin peaks at 90 min. The magnitude of the insulin response for the DIP participant is large compared to that of the SIP participant, more than doubling peak insulin from the approximately 300 μ U/mL in the SIP participant to approximately 700 μ U/mL in the DIP participant. In addition, the DIP participant exhibits an insufficient initial insulin response, an extended period of hyperglycemia, and an excursion below the basal glucose level to a nadir glucose level of 58 mg/dl of glucose, all indicators of poor control of central metabolism. The DIP participant is one a subset of individuals in our cohort who exhibits a hypoglycemic response. Both participants show an increase in glycerol concentrations above basal levels after the glucose nadir.

### Dynamics of glucose insulin action are delayed relative to dynamics of glycerol insulin action

**FIGURE 4:**
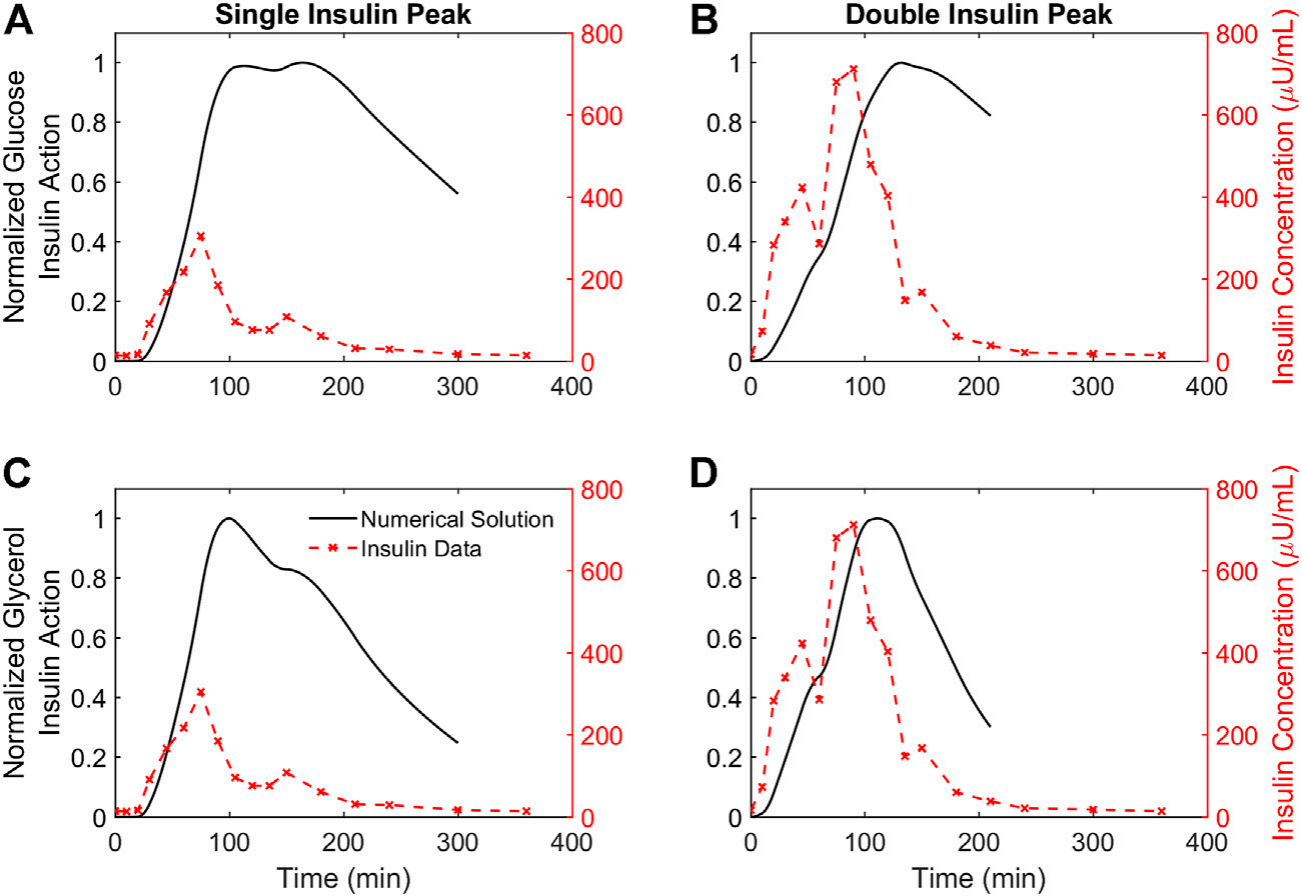
Time courses of insulin action on glucose and glycerol for two representative participants. **(A,B)**. The time course of insulin action on glucose plotted against insulin concentrations for two representative participants demonstrating a single insulin peak **(A)** and a double insulin peak **(B)**, respectively. **(C,D)**. The time course of insulin action on glycerol plotted against insulin concentrations for the same two representative participants. All insulin action concentrations are normalized by their maximum value. Insulin concentrations not normalized, and the DIP participant has higher insulin secretion compared to the SIP participant.

Each simulated glucose and glycerol profile has a corresponding insulin action profile. Insulin action profiles for the representative participants are shown in Figure 4. Both glucose and glycerol insulin action time traces rely on the same insulin concentration time series as a forcing function, but distinct dynamics for glucose and glycerol in response to insulin give rise to qualitatively different insulin action time traces. For both individuals, the glucose insulin action time trace shows a greater delay relative to the insulin time trace while the dynamics of the glycerol insulin action time trace follow insulin dynamics more closely. This observation that glucose insulin action has a greater delay relative to changing insulin concentration than the glycerol insulin action is consistent throughout the population and can be quantified using several metrics.

**FIGURE 5:**
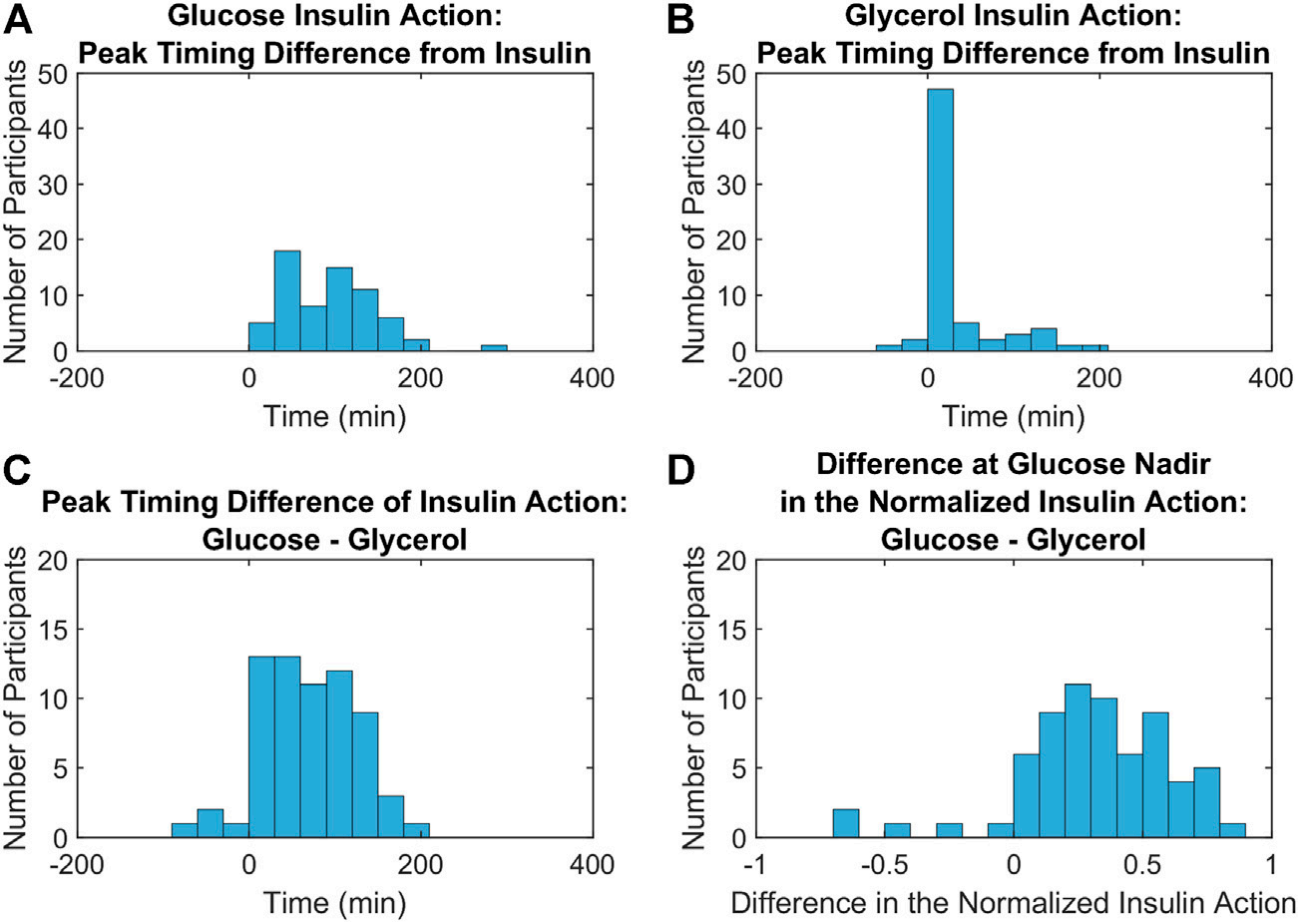
Metrics comparing the dynamics of insulin action on glucose and glycerol across all participants. **(A,B)**. Histograms of the differences between glucose **(A)** and glycerol **(B)** insulin action peak timing from insulin peak timing show that insulin peaks are closer to glycerol insulin action peaks compared to glucose insulin action peaks (Wilcoxon signed rank test, *p* < 0.001). **(C)**. A histogram of the differences between glucose and glycerol insulin action peak timing show that this difference is significantly greater than 0 (Student’s t-test, *p* < 0.001, 95% confidence interval: 67.38 ± 13.52), indicating that peak glucose insulin action occurs at a later time compared to peak glycerol insulin action. **(D)**. A histogram of the differences between normalized insulin actions for glucose and glycerol at the glucose nadir shows that the normalized insulin action for glucose is greater than the normalized insulin action for glycerol at this time point (Student’s t-test, *p* < 0.001, 95% confidence interval: 0.3120 ± 0.0736) and indicates that insulin action on glucose has stronger relative action at the glucose nadir.

The results from three metrics comparing distinct features of the insulin action profiles for glucose and glycerol in all participants are depicted in the histograms in Figure 5. The differences between glucose insulin action and insulin peak timing are larger and more variable compared to the differences between glycerol insulin action and insulin peak timing (Wilcoxon signed rank test, *p* < 0.001) reflecting the relatively later timing of the glucose insulin action peak (Figures 5A,B **)**. This relatively later timing of glucose insulin action is also seen in the difference in the timing of insulin action peaks for glucose and glycerol, where the glycerol insulin action peak time is subtracted from the glucose insulin action peak time (Figure 5C). The glycerol insulin action peak time was determined to be earlier compared to the glucose insulin action peak time with a difference between peak times significantly different from 0 (Student’s t-test, *p* < 0.001, 95% confidence interval: 67.38 ± 13.52). The normalized glucose insulin action is greater than the normalized glycerol insulin action at the glucose concentration nadir (Figure 5D). The difference in normalized insulin action was positive and significantly different from 0 (Student’s t-test, *p* < 0.001, 95% confidence interval: 0.3120 ± 0.0736). This difference indicates that glycerol insulin action terminates earlier compared to glucose insulin action relative to the timing of the glucose excursion. All of these metrics suggest that the timing of insulin action differs between tissues: glycerol insulin action on adipose tissue initiates and terminates earlier relative to glucose insulin action on hepatic tissue and muscle.

### Differences in the insulin action time constant

**FIGURE 6:**
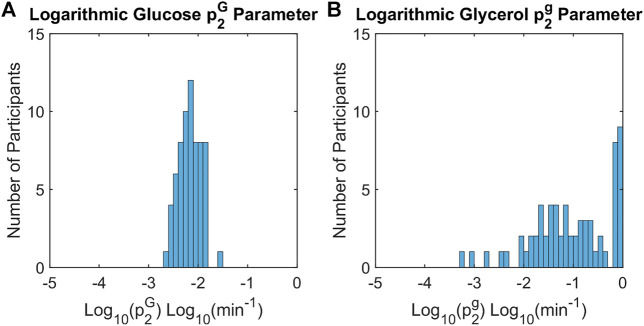
Histograms of insulin action time constants for glucose and glycerol across all participants. The time constants for insulin action on glucose, $p_{2}^{G}$ , **(A)** are consistently smaller than the time constants for insulin action on glycerol, $p_{2}^{g}$ , **(B)** (Wilcoxon signed rank test, *p* < 0.001). This indicates that the time course of insulin action on glucose is more delayed than the time course of insulin action on glycerol relative to insulin concentration data.

For glucose and glycerol insulin action models, the insulin action time constant parameters, $p_{2}^{G}$ and $p_{2}^{g}$ , respectively, govern the dynamics of insulin action. As the insulin action time constant parameters approach one, the insulin action curve approaches the plasma insulin curve. When the distributions of $p_{2}^{G}$ and $p_{2}^{g}$ were compared across all participants, the $p_{2}^{g}$ values for the glycerol model were much greater and were distributed across the range 0–1. To evaluate the effect of $p_{2}^{G}$ and $p_{2}^{g}$ on each model, the parameters were base 10 log transformed and compared. The distribution of the log transformed $p_{2}^{G}$ and $p_{2}^{g}$ values in all participants are shown in Figure 6. The estimates of the log-transformed parameters were significantly different (Wilcoxon signed rank test, *p* < 0.001) and show a distinct difference in magnitude with $p_{2}^{g}$ approximately two orders larger in magnitude than $p_{2}^{G}$ . The difference in estimated glycerol $p_{2}^{g}$ and glucose $p_{2}^{G}\;$ parameters indicates that insulin has a more immediate effect on glycerol insulin action than on glucose insulin action.

### Summary of differences in insulin action dynamics

**FIGURE 7:**
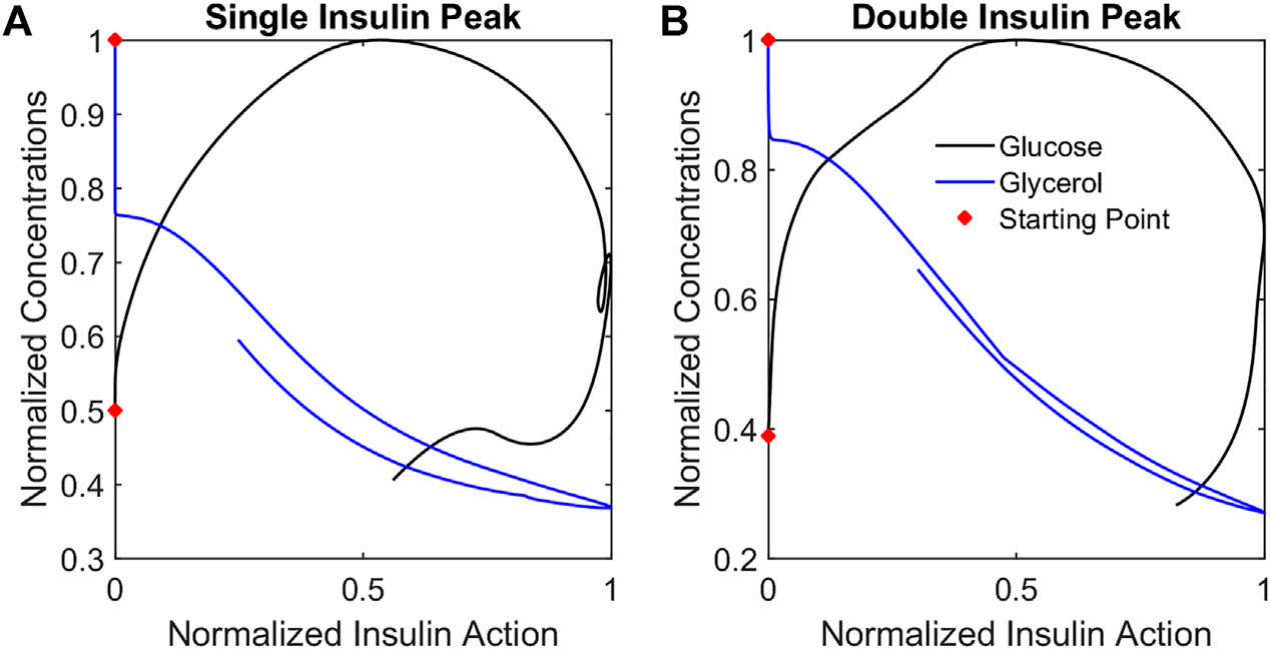
Metabolite phase plane trajectories summarize qualitative differences in glucose and glycerol dynamics relative to insulin action. Plotting normalized metabolite concentrations against normalized insulin action concentrations for the representative participants SIP **(A)** and DIP **(B)** reveals that glycerol concentrations change in a diagonal out-and-back pattern while the glucose concentrations change in a cyclic clockwise pattern reflecting the different dynamics of the responses.

To illustrate how insulin action changes relative to each metabolite, trajectories were considered in the metabolite-insulin action phase plane. Phase planes for each representative participant are shown in Figure 7. In each phase plane, the insulin action and metabolite were normalized by their maximum value. The phase planes show that changes in glycerol tracked more closely with changes in glycerol insulin action compared to changes in glucose and glucose insulin action. Specifically, the trajectory for the glycerol model showed an out and back diagonal path with glycerol and glycerol insulin action changing together. By contrast, the trajectory for the glucose model showed a cyclic path reflecting a time lag in changes in glucose insulin action relative to changes in glucose concentration.

## Discussion

### Summary of results

This study introduced a model of interacting glycerol and insulin dynamics in response to an OGTT and compared the dynamics of insulin acting on glucose and glycerol in a population of adolescent girls with obesity and with or without PCOS. To our knowledge, this glycerol model is the first mathematical model to describe interactions between glycerol and insulin dynamics. It successfully simulated glycerol concentration data over time from the ingestion of the drink to the post-excursion glucose nadir, and it demonstrated a suppression in glycerol concentrations in response to insulin action. Comparison of results from the glycerol model to results from OMM simulations of glucose and insulin dynamics showed that the dynamics of insulin action on glucose were delayed when compared to the dynamics of insulin action on glycerol.

### Differential dynamics for glucose and glycerol in adolescent girls

We quantified the dynamics of insulin action on glucose and glycerol based on model parameters and characteristics of the modeled insulin action using several metrics. All of these metrics showed that the dynamics of insulin action on glucose were delayed relative to the dynamics of insulin action on glycerol during the OGTT, and distinct representations of insulin action on glucose and glycerol were necessary to describe the metabolite data from our adolescent cohort.

Although we represent adipose metabolism through glycerol instead of FFA, the difference in dynamics we observe for insulin acting on glucose compared to insulin acting on glycerol likely reflects the extreme IR with compensatory hyperinsulinemia in our adolescent cohort. Our cohort has a significant degree of IR, accompanied by impaired glucose tolerance, with an average two-hour glucose measurement ≥ 140 mg/dl. Low insulin sensitivity suggests a slower insulin response, possibly increasing the delay in insulin action on the glucose system compared to the action of insulin on the glycerol system. The delayed timing of the insulin peaks in our cohort reflects extreme IR consistent with similar populations of adolescents with dysmetabolism (Cree-Green et al., 2018a; RISE Consortium, 2018). In normoglycemic non-obese youth, peak insulin concentrations occur at 30 min post drink, while the insulin peak is at 120 min in adolescents with prediabetes and diabetes (RISE Consortium, 2018; Tommerdahl et al., 2021). Our cohort has an insulin peak at 84 ± 47 min. However, the higher insulin concentrations required as a result of IR may also play a role in the observed delay of insulin action on the glucose system. The average peak insulin concentration for a healthy adolescent insulin profile is approximately 55 μ U/mL (Tommerdahl et al., 2021). The individuals in our cohort have an average peak insulin concentration of 361 μ U/mL. Whereas the insulin concentration needed to suppress lipolysis in this population, 40–50 μ U/mL, is reached quickly after consuming the drink, there is a much longer delay associated with reaching the peak insulin concentration which drives maximal glucose uptake (Cree-Green et al., 2016).

Adolescents have different metabolic characteristics compared to adults due to pubertally-mediated changes in insulin sensitivity, which present in addition to effects of obesity (RISE Consortium, 2018). Growth hormone alters both lipolysis and glucose metabolism, reducing insulin sensitivity in muscle and peripheral tissue, with concentrations peaking during the rapid growth phase of puberty (Moller and Jorgensen, 2009; Kim and Park, 2017). Growth hormone may preferentially influence IR in glucose metabolism compared to adipose metabolism producing a distinct metabolic phenotype in adolescents compared to phenotypes where IR is induced by other metabolic pathways. A tissue-specific difference in IR in adolescents could produce differential metabolic dynamics and is consistent with our findings that data in this cohort requires separate models for insulin action on glucose and glycerol during an OGTT.

By contrast, Periwal and colleagues described glucose and FFA dynamics in an IM-FSIVGTT and a mixed meal tolerance test (MMTT) in African American and Caucasian premenopausal women using a single model with one form of insulin action (Periwal et al., 2008; Li et al., 2016). In addition to the dissimilarities between study populations, distinct dynamics of glucose, insulin, glycerol and FFA among experimental protocols may contribute to the differences in our findings. In an IM-FSIVGTT, plasma glucose concentrations peak at the beginning of the protocol, and the initial early peak in insulin reflects the injection of exogenous insulin and may interact with the endogenous glucose-insulin dynamics and diminish endogenous insulin release. In an OGTT, ingested glucose is slowly absorbed and typically peaks at least 20 min after the administration of the drink (Cree-Green et al., 2018b; RISE Consortium, 2018); endogenous insulin is released in response to increased plasma glucose concentrations and acts on glycerol and glucose in a concentration-dependent manner. In an MMTT, the absorbance of glucose is slower compared to an OGTT due to the presence of fat and protein (Li et al., 2016).

Thus, although, the glucose and FFA model captured the dynamics of two very disparate methods of increasing glucose and insulin in an adult population, the temporality of changes in glucose, insulin, and FFA were similar within each protocol (all fast in an IM-FSIVGTT and all slow in a MMTT). By contrast, an OGTT may highlight distinct dynamics between adipose and glucose metabolism by producing physiologic interactions between glucose and endogenous insulin dynamics in the context of glucose absorbance, that is, slower compared to an IM-FSIVGTT and faster compared to an MMTT. Thus, differences in study populations and protocols likely contributed to the differences in temporality and rate of changes between glucose, insulin, and glycerol and necessitated distinct representations of insulin action on glucose and glycerol in our study compared to previous work with FFAs (Periwal et al., 2008; Li et al., 2016).

### Possible physiologic basis for difference in dynamics

Insulin regulation of the metabolic pathways for glucose and glycerol occurs through distinct mechanisms. The elevation of glucose concentration triggers the release of insulin. The insulin then acts so that glucose concentrations decrease back to basal levels. When glucose concentrations return to normal, insulin secretion also decreases. Thus, the interaction between glucose and insulin is bidirectional. Conversely, the interaction between glycerol and insulin is unidirectional. Insulin induces the suppression of lipolysis by regulating the activity of hormone sensitive lipase (Stralfors and Honnor, 1989; Arner, 2001). When insulin concentrations decrease, activation of hormone sensitive lipase stops, and glycerol concentrations increase. However, glycerol concentration has no effect on insulin concentration.

### Limitations

This model makes several simplifying assumptions about glycerol biochemistry. First, although we expect lipolysis to be the primary source of glycerol in our protocol, glycolysis may play a role (Rotondo et al., 2019). Second, the structure of this glycerol model assumes that the maximum lipolysis rate occurs in the initial fasted state, and, therefore, it cannot describe rebounds in glycerol concentrations above basal levels. In many participants in our cohort (both SIP and DIP), glycerol concentrations post-suppression rose above basal levels, suggesting the involvement of other metabolic pathways. This post-suppression rebound was particularly pronounced in the approximately 10% of participants demonstrating reactive hypoglycemia (RHG) (Ware et al., 2022). Hypoglycemia is characterized as a condition where blood sugar falls below 60 mg/dl, resulting in warning symptoms and the secretion of counterregulatory hormones working to rapidly increase blood sugar levels (Desouza et al., 2010; Casertano et al., 2021; Ware et al., 2022). Along with glucagon, catecholamines are released during a RHG response, stimulating lipolysis (Fanelli et al., 2020). The current glycerol model does not account for these additional metabolic pathways, so we truncated the data at the glucose nadir to avoid trying to represent two distinct physiological conditions (the initial glucose excursion and the recovery of lipolysis above basal rates) with a single set of parameters. Future work should consider extensions of the glycerol model that account for the counterregulatory response.

There are several additional limitations to this study. This model was developed in a highly IR population of adolescent girls with a high incidence of non-alcoholic fatty liver disease (NAFLD), a condition associated with adipose dysmetabolism. Application of the model to data from healthy populations as well as other IR or dysglycemic populations is important to verify the generalizability of this glycerol-insulin model to the range of dynamics associated with adipose metabolism. For example, in a healthy individual, glycerol may be suppressed earlier in response to a smaller plasma insulin peak.

### Summary and implications

In summary, we have proposed a novel differential equations-based model of interactions between glycerol and insulin dynamics that provides a better understanding of glycerol dynamics relative to other metabolic processes like glucose metabolism. In addition, this model demonstrates that during an OGTT, insulin action on glucose is more delayed compared to insulin action on glycerol in our cohort of IR adolescent girls. Although tissue-specific actions of insulin are known to be concentration dependent, to our knowledge this is the first study to establish a difference in the dynamics of distinct insulin actions. Future work examining the mechanisms implicated in this difference and the significance of altered relative glycerol and glucose dynamics to metabolic disease development and progression is needed to alleviate the growing burden of metabolic dysregulation.

## Data Availability

The datasets presented in this article are not readily available because an appropriate institutional data sharing agreement is required. Requests to access the datasets should be directed to Melanie Cree-Green, Melanie.Green@childrenscolorado.org.

## References

[B1] Adler-Wailes D. C. Periwal V. Ali A. H. Brady S. M. McDuffie J. R. Uwaifo G. I. (2013). Sex-associated differences in free fatty acid flux of obese adolescents. J. Clin. Endocrinol. Metab. 98 (4), 1676–1684. 10.1210/jc.2012-3817 23450055 PMC3615213

[B2] Aguilar M. Bhuket T. Torres S. Liu B. Wong R. J. (2015). Prevalence of the metabolic syndrome in the United States, 2003-2012. JAMA 313 (19), 1973–1974. 10.1001/jama.2015.4260 25988468

[B3] Ajmera I. Swat M. Laibe C. Le Novere N. Chelliah V. (2013). The impact of mathematical modeling on the understanding of diabetes and related complications. CPT. Pharmacometrics Syst. Pharmacol. 2, e54. 10.1038/psp.2013.30 23842097 PMC3731829

[B4] American Diabetes A. (2020). 13. Children and adolescents: Standards of medical care in diabetes-2020. Diabetes Care 43 (1), S163–S182. 10.2337/dc20-S013 31862756

[B5] Arner P. (2001). Free fatty acids - do they play a central role in type 2 diabetes? Diabetes Obes. Metab. 3, 11–19. 10.1046/j.1463-1326.2001.00031.x 11683854

[B6] Arner P. (2002). Insulin resistance in type 2 diabetes: role of fatty acids. Diabetes. Metab. Res. Rev. 18 (2), S5–S9. 10.1002/dmrr.254 11921432

[B7] Arner P. Rydén M. (2015). Fatty acids, obesity and insulin resistance. Obes. Facts 8 (2), 147–155. 10.1159/000381224 25895754 PMC5644864

[B8] Bartlette K. Carreau A. M. Xie D. Garcia-Reyes Y. Rahat H. Pyle L. (2021). Oral minimal model-based estimates of insulin sensitivity in obese youth depend on oral glucose tolerance test protocol duration. Metabol. Open 9, 100078. 10.1016/j.metop.2021.100078 33511337 PMC7817496

[B9] Bergman R. N. Ider Y. Z. Bowden C. R. Cobelli C. (1979). Quantitative estimation of insulin sensitivity. Am. J. Physiol. 236 (6), E667–E677. 10.1152/ajpendo.1979.236.6.E667 443421

[B10] Bergman R. N. (1989). Lilly lecture 1989. Toward physiological understanding of glucose tolerance. Minimal-model approach. Diabetes 38 (12), 1512–1527. 10.2337/diab.38.12.1512 2684710

[B11] Bergman RNB C. R. Cobelli C. (1981). “The Minimal Model approach to quantification of factors controlling glucose disposal in man,” in Carbohydrate metabolism. Editor CCB R. N. (John Wiley & Sons), 13, 269–296.

[B12] Casertano A. Rossi A. Fecarotta S. Rosanio F. M. Moracas C. Di Candia F. (2021). An overview of hypoglycemia in children including a comprehensive practical diagnostic flowchart for clinical use. Front. Endocrinol. 12, 684011. 10.3389/fendo.2021.684011 PMC8366517 34408725

[B13] Chooi Y. C. Ding C. Magkos F. (2019). The epidemiology of obesity. Metabolism. 92, 6–10. 10.1016/j.metabol.2018.09.005 30253139

[B14] Cobelli C. Dalla Man C. Toffolo G. Basu R. Vella A. Rizza R. (2014). The oral minimal model method. Diabetes 63 (4), 1203–1213. 10.2337/db13-1198 24651807 PMC4179313

[B15] RISE Consortium and Investigators, R. C. (2019). Effects of treatment of impaired glucose tolerance or recently diagnosed type 2 diabetes with metformin alone or in combination with insulin glargine on beta-cell function: Comparison of responses in youth and adults. Diabetes 68 (8), 1670–1680. 10.2337/db19-0299 31178433 PMC6692818

[B16] RISE Consortium (2018). Metabolic contrasts between youth and adults with impaired glucose tolerance or recently diagnosed type 2 diabetes: I. Observations using the hyperglycemic clamp. Diabetes Care 41 (8), 1696–1706. 10.2337/dc18-0244 29941497 PMC6054493

[B17] Conte C. Fabbrini E. Kars M. Mittendorfer B. Patterson B. W. Klein S. (2012). Multiorgan insulin sensitivity in lean and obese subjects. Diabetes Care 35 (6), 1316–1321. 10.2337/dc11-1951 22474039 PMC3357234

[B18] Centers for Disease Control and Prevention (2020). National diabetes statistics report. Atlanta, GA: Centers for Disease Control and Prevention, US Department of Health and Human Services.

[B19] Coppack S. W. Persson M. Judd R. L. Miles J. M. (1999). Glycerol and nonesterified fatty acid metabolism in human muscle and adipose tissue *in vivo* . Am. J. Physiol. 276 (2), E233–E240. 10.1152/ajpendo.1999.276.2.E233 9950781

[B20] Cree-Green M. Bergman B. C. Cengiz E. Fox L. A. Hannon T. S. Miller K. (2019). Metformin improves peripheral insulin sensitivity in youth with type 1 diabetes. J. Clin. Endocrinol. Metab. 104 (8), 3265–3278. 10.1210/jc.2019-00129 30938764 PMC6584133

[B21] Cree-Green M. Bergman B. C. Coe G. V. Newnes L. Baumgartner A. D. Bacon S. (2016). Hepatic steatosis is common in adolescents with obesity and PCOS and relates to De novo lipogenesis but not insulin resistance. Obes. (Silver Spring) 24 (11), 2399–2406. 10.1002/oby.21651 PMC5117819 27804265

[B22] Cree-Green M. Cai N. Thurston J. E. Coe G. V. Newnes L. Garcia-Reyes Y. (2018). Using simple clinical measures to predict insulin resistance or hyperglycemia in girls with polycystic ovarian syndrome. Pediatr. Diabetes 19 (8), 1370–1378. 10.1111/pedi.12778 30246333 PMC6400639

[B23] Cree-Green M. Wiromrat P. Stuppy J. J. Thurston J. Bergman B. C. Baumgartner A. D. (2019). Youth with type 2 diabetes have hepatic, peripheral, and adipose insulin resistance. Am. J. Physiol. Endocrinol. Metab. 316 (2), E186–E195. 10.1152/ajpendo.00258.2018 30562061 PMC6397366

[B24] Cree-Green M. Xie D. Rahat H. Garcia-Reyes Y. Bergman B. C. Scherzinger A. (2018). Oral glucose tolerance test glucose peak time is most predictive of prediabetes and hepatic steatosis in obese girls. J. Endocr. Soc. 2 (6), 547–562. 10.1210/js.2018-00041 29942919 PMC6007246

[B25] Dalla Man C. Caumo A. Cobelli C. (2002). The oral glucose minimal model: Estimation of insulin sensitivity from a meal test. IEEE Trans. Biomed. Eng. 49 (5), 419–429. 10.1109/10.995680 12002173

[B26] Desouza C. V. Bolli G. B. Fonseca V. (2010). Hypoglycemia, diabetes, and cardiovascular events. Diabetes Care 33 (6), 1389–1394. 10.2337/dc09-2082 20508232 PMC2875462

[B27] Diniz Behn C. Jin E. S. Bubar K. Malloy C. Parks E. J. Cree-Green M. (2020). Advances in stable isotope tracer methodology part 1: hepatic metabolism *via* isotopomer analysis and postprandial lipolysis modeling. J. Investig. Med. 68 (1), 3–10. 10.1136/jim-2019-001109 PMC7372575 31554675

[B28] Fanelli C. G. Lucidi P. Bolli G. B. Porcellati F. (2020). Hypoglycemia. Springer International Publishing, 615–652.

[B29] Group T. S. Bjornstad P. Drews K. L. Caprio S. Gubitosi-Klug R. Nathan D. M. (2021). Long-term complications in youth-onset type 2 diabetes. N. Engl. J. Med. Overseas. Ed. 385 (5), 416–426. 10.1056/nejmoa2100165 PMC8697255 34320286

[B30] Ha J. Satin L. S. Sherman A. S. (2016). A mathematical model of the pathogenesis, prevention, and reversal of type 2 diabetes. Endocrinology 157 (2), 624–635. 10.1210/en.2015-1564 26709417 PMC4733125

[B31] Hirode G. Wong R. J. (2020). Trends in the prevalence of metabolic syndrome in the United States, 2011-2016. JAMA 323 (24), 2526–2528. 10.1001/jama.2020.4501 32573660 PMC7312413

[B32] Imperatore G. Boyle J. P. Thompson T. J. Case D. Dabelea D. Hamman R. F. (2012). Projections of type 1 and type 2 diabetes burden in the U.S. population aged <20 years through 2050: dynamic modeling of incidence, mortality, and population growth. Diabetes Care 35 (12), 2515–2520. 10.2337/dc12-0669 23173134 PMC3507562

[B33] Jensen M. D. (1999). Regional glycerol and free fatty acid metabolism before and after meal ingestion. Am. J. Physiol. 276 (5), E863–E869. 10.1152/ajpendo.1999.276.5.E863 10329980

[B34] Kelly T. Yang W. Chen C. S. Reynolds K. He J. (2008). Global burden of obesity in 2005 and projections to 2030. Int. J. Obes. 32 (9), 1431–1437. 10.1038/ijo.2008.102 18607383

[B35] Kim S. H. Park M. J. (2017). Effects of growth hormone on glucose metabolism and insulin resistance in human. Ann. Pediatr. Endocrinol. Metab. 22 (3), 145–152. 10.6065/apem.2017.22.3.145 29025199 PMC5642081

[B36] Landau B. R. (1999). Glycerol production and utilization measured using stable isotopes. Proc. Nutr. Soc. 58 (4), 973–978. 10.1017/s0029665199001287 10817165

[B37] Levine J. A. Han J. M. Wolska A. Wilson S. R. Patel T. P. Remaley A. T. (2020). Associations of GlycA and high-sensitivity C-reactive protein with measures of lipolysis in adults with obesity. J. Clin. Lipidol. 14 (5), 667–674. 10.1016/j.jacl.2020.07.012 32863171 PMC7642018

[B38] Li Y. Chow C. C. Courville A. B. Sumner A. E. Periwal V. (2016). Modeling glucose and free fatty acid kinetics in glucose and meal tolerance test. Theor. Biol. Med. Model. 13, 8. 10.1186/s12976-016-0036-3 26934990 PMC4776401

[B39] Magkos F. Fabbrini E. Conte C. Patterson B. W. Klein S. (2012). Relationship between adipose tissue lipolytic activity and skeletal muscle insulin resistance in nondiabetic women. J. Clin. Endocrinol. Metab. 97 (7), E1219–E1223. 10.1210/jc.2012-1035 22492868 PMC3387393

[B40] Mayer-Davis E. J. Lawrence J. M. Dabelea D. Divers J. Isom S. Dolan L. (2017). Incidence trends of type 1 and type 2 diabetes among youths, 2002-2012. N. Engl. J. Med. 376 (15), 1419–1429. 10.1056/NEJMoa1610187 28402773 PMC5592722

[B41] Moller N. Jorgensen J. O. (2009). Effects of growth hormone on glucose, lipid, and protein metabolism in human subjects. Endocr. Rev. 30 (2), 152–177. 10.1210/er.2008-0027 19240267

[B42] Periwal V. Chow C. C. Bergman R. N. Ricks M. Vega G. L. Sumner A. E. (2008). Evaluation of quantitative models of the effect of insulin on lipolysis and glucose disposal. Am. J. Physiol. Regul. Integr. Comp. Physiol. 295 (4), R1089–R1096. 10.1152/ajpregu.90426.2008 18685069 PMC2576090

[B43] Petersen M. C. Shulman G. I. (2018). Mechanisms of insulin action and insulin resistance. Physiol. Rev. 98 (4), 2133–2223. 10.1152/physrev.00063.2017 30067154 PMC6170977

[B44] Picchini U. De Gaetano A. Panunzi S. Ditlevsen S. Mingrone G. (2005). A mathematical model of the euglycemic hyperinsulinemic clamp. Theor. Biol. Med. Model. 2 (1), 44. 10.1186/1742-4682-2-44 16269082 PMC1291408

[B45] Possik E. Schmitt C. Al-Mass A. Bai Y. Cote L. Morin J. (2022). Phosphoglycolate phosphatase homologs act as glycerol-3-phosphate phosphatase to control stress and healthspan in *C. elegans* . Nat. Commun. 13 (1), 177. 10.1038/s41467-021-27803-6 35017476 PMC8752807

[B46] Ramos-Roman M. A. Lapidot S. A. Phair R. D. Parks E. J. (2012). Insulin activation of plasma nonesterified fatty acid uptake in metabolic syndrome. Arterioscler. Thromb. Vasc. Biol. 32 (8), 1799–1808. 10.1161/ATVBAHA.112.250019 22723441 PMC3417322

[B47] Reshef L. Olswang Y. Cassuto H. Blum B. Croniger C. M. Kalhan S. C. (2003). Glyceroneogenesis and the triglyceride/fatty acid cycle. J. Biol. Chem. 278 (33), 30413–30416. 10.1074/jbc.R300017200 12788931

[B48] Ronald Kahn C. (1978). Insulin resistance, insulin insensitivity, and insulin unresponsiveness: A necessary distinction. Metabolism. 27 (12), 1893–1902. 10.1016/s0026-0495(78)80007-9 723640

[B49] Rotondo F. Ho-Palma A. C. Romero M. D. M. Remesar X. Fernandez-Lopez J. A. Alemany M. (2019). Higher lactate production from glucose in cultured adipose nucleated stromal cells than for rat adipocytes. Adipocyte 8 (1), 61–76. 10.1080/21623945.2019.1569448 30676233 PMC6768231

[B50] Roy A. Parker R. S. (2006). Dynamic modeling of free fatty acid, glucose, and insulin: An extended "minimal model. Diabetes Technol. Ther. 8 (6), 617–626. 10.1089/dia.2006.8.617 17109593

[B51] Sondergaard E. Espinosa De Ycaza A. E. Morgan-Bathke M. Jensen M. D. (2017). How to measure adipose tissue insulin sensitivity. J. Clin. Endocrinol. Metab. 102 (4), 1193–1199. 10.1210/jc.2017-00047 28323973 PMC5460729

[B52] Steinberg D. Vaughan M. Margolis S. Price H. Pittman R. (1961). Studies of triglyceride biosynthesis in homogenates of adipose tissue. J. Biol. Chem. 236 (6), 1631–1637. 10.1016/s0021-9258(19)63276-x

[B53] Stralfors P. Honnor R. C. (1989). Insulin-induced dephosphorylation of hormone-sensitive lipase. Correlation with lipolysis and cAMP-dependent protein kinase activity. Eur. J. Biochem. 182 (2), 379–385. 10.1111/j.1432-1033.1989.tb14842.x 2661229

[B54] Thomaseth K. Brehm A. Pavan A. Pacini G. Roden M. (2014). Modeling glucose and free fatty acid kinetics during insulin-modified intravenous glucose tolerance test in healthy humans: role of counterregulatory response. Am. J. Physiol. Regul. Integr. Comp. Physiol. 307 (3), R321–R331. 10.1152/ajpregu.00314.2013 24848363

[B55] Tommerdahl K. L. Brinton J. T. Vigers T. Cree-Green M. Zeitler P. S. Nadeau K. J. (2021). Delayed glucose peak and elevated 1-hour glucose on the oral glucose tolerance test identify youth with cystic fibrosis with lower oral disposition index. J. Cyst. Fibros. 20 (2), 339–345. 10.1016/j.jcf.2020.08.020 32928701 PMC7947033

[B56] Utzschneider K. M. Tripputi M. T. Kozedub A. Barengolts E. Caprio S. Cree-Green M. (2021). Differential loss of beta-cell function in youth vs. adults following treatment withdrawal in the Restoring Insulin Secretion (RISE) study. Diabetes Res. Clin. Pract. 178, 108948. 10.1016/j.diabres.2021.108948 34274407 PMC8628318

[B57] Utzschneider K. M. Tripputi M. T. Kozedub A. Mather K. J. Nadeau K. J. Edelstein S. L. (2020). β-cells in youth with impaired glucose tolerance or early type 2 diabetes secrete more insulin and are more responsive than in adults. Pediatr. Diabetes 21 (8), 1421–1429. 10.1111/pedi.13113 32902875 PMC7642023

[B58] Ware M. Carreau A. Garcia-Reyes Y. Rahat H. Diniz Behn C. Cree-Green M. (2022). Reactive hypoglycemia following a sugar challenge is accompanied by higher insulin in adolescent girls with obesity. J. Investig. Med. 70, 112-337.

[B59] Wolfe R. R. Chinkes D. L. (2005). Isotope tracers in metabolic research: Principles and practice of kinetic analysis. 2nd ed. (Hoboken, N.J.: Wiley-Liss), 474. vii.

[B60] Young L. H. Periwal V. (2016). Metabolic scaling predicts posthepatectomy liver regeneration after accounting for hepatocyte hypertrophy. Liver Transpl. 22 (4), 476–484. 10.1002/lt.24392 26709233 PMC4809762

[B61] Zawadzki JD A. (1992). “Diagnostic criteria for polycystic ovary syndrome: towards a rational approach,” in Polycystic ovary syndrome (Boston: Blackwell Scientific Publications), 39–50.

